# Validity of Reporting Oxygen Uptake Efficiency Slope from Submaximal Exercise Using Respiratory Exchange Ratio as Secondary Criterion

**DOI:** 10.1155/2012/874020

**Published:** 2012-05-14

**Authors:** Wilby Williamson, Jonathan Fuld, Kate Westgate, Karl Sylvester, Ulf Ekelund, Soren Brage

**Affiliations:** ^1^Medical Research Council Epidemiology Unit, Institute of Metabolic Science, Box 285, Addenbrooke's Hospital, Cambridge CB2 0QQ, UK; ^2^Department of Respiratory Medicine, Cambridge University Hospitals, NHS Foundation Trust, Cambridge CB2 0QQ, UK

## Abstract

*Background*. Oxygen uptake efficiency slope (OUES) is a reproducible, objective marker of cardiopulmonary function. OUES is reported as being relatively independent of exercise intensity. Practical guidance and criteria for reporting OUES from submaximal tests has not been established. *Objective*. Evaluate the use of respiratory exchange ratio (RER) as a secondary criterion for reporting OUES. *Design*. 100 healthy volunteers (53 women) completed a ramped treadmill protocol to exhaustive exercise. OUES was calculated from data truncated to RER levels from 0.85 to 1.2 and compared to values generated from full test data. Results. Mean (sd) OUES from full test data and data truncated to RER 1.0 and RER 0.9 was 2814 (718), 2895 (730), and 2810 (789) mL/min per 10-fold increase in VE, respectively. Full test OUES was highly correlated with OUES from RER 1.0 (*r* = 0.9) and moderately correlated with OUES from RER 0.9 (*r* = 0.79). *Conclusion*. OUES values peaked in association with an RER level of 1.0. Sub-maximal OUES values are not independent of exercise intensity. There is a significant increase in OUES value as exercise moves from low to moderate intensity. RER can be used as a secondary criterion to define this transition.

## 1. Introduction

Exercise testing allows quantification of cardiopulmonary function providing valuable diagnostic and prognostic data. Peak and maximal cardiopulmonary exercise testing are gold standard modalities [1]. However, in clinical practice and field research reporting peak exercise parameters can be compromised by compliance and feasibility. There are a number of extrinsic factors, including financial restraints and risk mitigation protocols that can restrict exercise testing to submaximal intensities. Exercise testing in large-scale population studies has to ensure high participant turnover and maintain safety in often nonclinical, potentially resource-depleted environments. To maintain safety and efficiency, termination criteria are within the moderate, nonmaximal exercise intensity range. Tests stopped within 80–90% of maximal heart rate or when respiratory exchange ratio (RER) reaches 0.9–1.0 [2, 3]. Terminating exercise within RER ranges of 0.9–1.0 places significant restrictions on the reporting of gas exchange data.

In clinical practice, the most frequently reported submaximal parameter is the ventilatory anaerobic threshold (VAT). However, reporting VAT is not without limitations, with potential for observer error or technical difficulties when defining values [4, 5]. These challenges are even more pronounced when using exercise tests with termination criteria of RER 0.9-1.0. Shorter test durations restrict the number of data points and intensity may not progress far enough beyond VAT to confidently report results using the recognised V-slope methods [6]. Under these conditions, it may also be more difficult to accommodate irregularities in breathing patterns. For example, hyperventilation that might be expected to settle as exercise progresses may compromise the reporting of graphical submaximal data points. These practical limitations provide an incentive to establish objective, reproducible submaximal gas exchange parameters with functional and prognostic value.

There are a number of potential parameters that can be reported from submaximal gas exchange data ranging from regressions of ventilation versus carbon dioxide exhalation to measures derived from oxygen uptake [7, 8]. The oxygen uptake efficiency slope (OUES) is a regression-derived parameter from the relationship between log-transformed minute ventilation (VE) and oxygen uptake (VO_2_), with the coefficient “*a*” from the regression VO_2_ = *a*  logVE + *b* being defined as the OUES. The coefficient “*a*” represents the rate of change in VO_2_ in response to VE [9]. If an individual achieves a higher VO_2_ with only a small increase in VE, this will produce a higher OUES and this is taken to represent more efficient oxygen uptake. OUES is regarded as an objective, reproducible marker of cardiopulmonary function calculated from sequential data points. Using sequential data points as opposed to time or intensity defined values has led to the suggestion that OUES represents a composite value for cardiopulmonary function inclusive of the physiological transition from low to vigorous intensity [9, 10].

In the context of maximal testing, OUES provides a similar marker of function and prognosis as Peak VO_2_ [11–13]. However, the evidence that OUES remains relatively stable across moderate-to-high intensity exercise has promoted acceptance of OUES as a valid submaximal measure of function and disease prognosis [10]. Baba and coworkers were first to report the use of OUES in cardiovascular populations and identify the relative stability of OUES in the final quartile of a maximal test [9]. Hollenberg et al. then confirmed these results; OUES reported from 75% of the completed test differed by less than 2% of the OUES calculated from the full test [12]. These seminal papers provided the template to establish reporting criterion for submaximal OUES values and facilitate expansion into clinical practice. However, translation into the clinical domain has been slow despite the literature continuing to grow in support of OUES as a functional and prognostic parameter during peak and maximal tests [13, 14]. The majority of studies continue to report OUES defined by percentage data acknowledging that there is a strong correlation between submaximal and full test results [15]. Lending support to the statement that OUES is relatively independent of exercise intensity but not defining reporting criterion. Pogliahgi et al. explored defining submaximal OUES with regards to percentages of predicted heart rate reserve [16]. Heart rate is commonly used in noninvasive exercise testing to define intensity but the variance and potential error, especially in the clinical sitting, is well established [17]. Overall, there has been minimal progression in the literature on defining how to practically use OUES with reference to submaximal testing. This is potentially limiting the expansion of OUES in both the clinical and research domains.

Clinically, there is rarely the luxury of collecting peak exercise data, and in the context of submaximal tests, it is difficult to define percentage efforts. The same limitation holds for researchers working with large populations where field testing is limited by feasibility to submaximal tests. In both of these contexts OUES could be an ideal parameter to report. The objective of the current study is to report the reliability of using RER as a reporting criterion for OUES, exploring the question of whether there is a submaximal threshold below which OUES is not valid or incurs significant error when compared to true maximal data.

## 2. Methods

### 2.1. Participants

A total of 100 participants were recruited from the Cambridge area (UK). Participants were free from cardiopulmonary and metabolic diseases. Ethical approval for the study was obtained from the local research ethics committee. All participants provided written, informed consent.

### 2.2. Study Procedure

Participants were asked to refrain from eating, drinking (except water), smoking, and vigorous exercise for at least 2 hours before they arrived at the laboratory. Height and weight of participants in light clothing were recorded using a rigid stadiometer and calibrated scales, respectively.

### 2.3. Treadmill Test

A Jaeger Oxycon Pro system was configured to control a motorized treadmill (HP Cosmo Pulsar 4.0). The treadmill protocol was a nonindividualised ramp protocol adapted from an original epidemiological study protocol [18]. The original protocol was extended to include a 4th phase to ensure participants exercised to an exhaustive intensity. *Phase 1 *(level walking) involved level walking with increasing speed (3 min at 3.2 km/h and then accelerating at 0.33 km·h^−1^ per min for the next 6 min), *phase 2 *(graded walking) consisted of brisk walking (5.2–5.8 km/h) with increasing gradient (at a rate of 1.7% increased gradient/min for 6 min), *phase 3 *(level running) involved level running with speed increasing from 9 to 12.5 km/h for 4.5 min (average acceleration of 0.78 km·h^−1^ per min), and phase 4 (uphill running) in which the gradient increased by 0.5% and the speed increased by 0.25 km·hr^−1^ every 15 seconds until exhaustion. Transition between *phases 2 *and *3 *was first a change in gradient by −10.2% over 30 seconds (now level), followed by a change in speed by 3.2 km/h over 30 seconds.

Continuous recording of respiratory gas exchange parameters was taken during the treadmill test and for 2 minutes during recovery after exercise test termination. Participants were asked to exercise until maximal exertion. Clinical indicators for terminating the treadmill test were onset of angina or angina-like symptoms or signs of poor perfusion including light-headedness, confusion, ataxia, pallor, cyanosis, nausea, or cold and clammy skin. In addition, tests were stopped following physical or verbal manifestations of severe fatigue, the volunteer requesting to stop despite verbal encouragement.

### 2.4. Respiratory Gas Analysis

Gas exchange data were acquired breath by breath and averaged over 20-second intervals for generation of graphic data and regression analysis for OUES and VE/VCO_2_ [7]. Peak VO_2_ and peak respiratory exchange ratio was expressed as the highest averaged values over sequential 30-second periods obtained from complete exercise data [11, 19]. Breath by breath averaging was expanded to 30 seconds in an attempt to reduce the effect of transient fluctuations in RER when calculating RER truncated OUES. RER data for each individual test was plotted against time to review trend. The VAT was determined by the V-slope method [6].

### 2.5. Determination of Oxygen Uptake Efficiency across Exercise Duration

OUES was calculated from complete and truncated gas exchange data according to a series of criteria.

1. Defined as percentile of the complete test. OUES calculated from data taken from the first 25%, 50%, and 75% of time defined test data.
2. Defined according to Ventilatory Anaerobic Threshold (VAT). Calculating OUES using data from start of test until time of VAT.
3. Defined according to increasing Respiratory Exchange Ratio (RER). OUES values were calculated from test data limited to RER ≤ 0.85, RER ≤ 0.90, RER ≤ 0.95, RER ≤ 1.00, RER ≤ 1.10, and RER ≤ 1.20.

### 2.6. Gas Exchange Reference Values

Mean Peak VO_2_, OUES, and VEVCO_2_ results were compared with predicted reference ranges accounting for age, weight, height, and sex [7, 11, 19].

Peak VO_2_ prediction equation:

Men: VO_2_max = [50.2 − (0.394 (age (yrs))],
Women: VO_2_max = [42.83 − (0.371 (age (yrs))].

OUES prediction equations:

Men: OUES [L/min/log(L/min)] = [−0.61 − 0.032 (age (yrs)) + 0.023(height (cm)) + 0.008 (weight (kg))],
Women: OUES [L/min/log(L/min)] = [−1.178 − 0.032 (age (yrs)) + 0.023 (height (cm)) + 0.008 (weight (kg))].

VE/VCO_2_ prediction equations:

Men: VE/VCO_2_ slope = 34.5 + 0.1 (age (yrs)) − 0.05 (height) (cm),
Women: VE/VCO_2_ slope = 35.5 + 0.1(age (yrs)) − 0.05(height) (cm).

### 2.7. Statistics

Statistical analysis was performed using StataIC 11 (Stata Corp LP, TX).

Summary statistics for continuous variables are expressed as means with standard deviation (+SD). Relative and absolute agreement between the different measures of OUES were reported via correlation and Bland-Altman agreement analysis (including root mean square error). When displayed graphically, mean values are presented with standard error bars. Subgroup analysis was performed using Student *t*-test and regression analysis.

## 3. Results

One hundred participants (53 women) underwent cardiopulmonary exercise testing (CPET) recording Peak VO_2_, VAT, OUES, and VE/VCO_2_ slope values. The group were healthy volunteers. The mean age was 41.4 yrs (range 22 to 65 yrs). Mean weight was 70.3 kg (range 44 to 109 kg) and mean height was 170.5 cm (range 144 to 195 cm).

Mean test duration was 1228 seconds (sd 152 s). Mean time to VAT was 821 seconds associated with an RER of 0.92 (sd 0.07). Only one individual passed the anaerobic threshold prior to 600 seconds. No participants were limited by clinical symptoms. Mean Peak VO_2_ was 39.8 (sd 8.8) mL/kg/min; mean Peak RER was 1.19 (sd 0.11). Mean OUES using all available test data was 2814 (sd 718) (mL/min/logVE); mean VE/VCO_2_ slope using all test data was 30.2 (sd 4.15). Exercise characteristics from the complete test are shown in Table 1. The results are consistent with high cardiovascular fitness within this sample; measured Peak VO_2_ was 131.5% of the expected value.

### 3.1. OUES Values during Cardiopulmonary Exercise Testing

**Figure 1:**
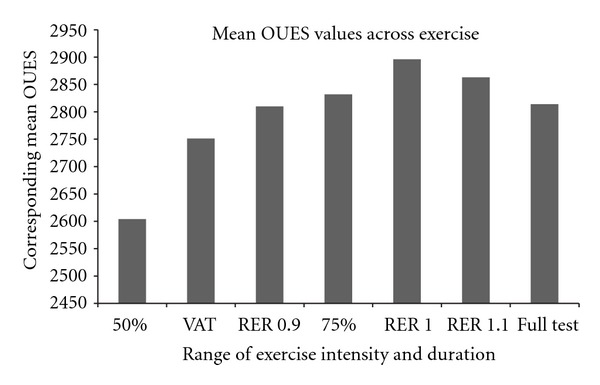
Mean OUES values between 50th percentile and full test defined by submaximal criteria. VAT Ventilatory Anaerobic Threshold, RER respiratory exchange ratio. Percentage of test defined by time to complete full test.

**Figure 2:**
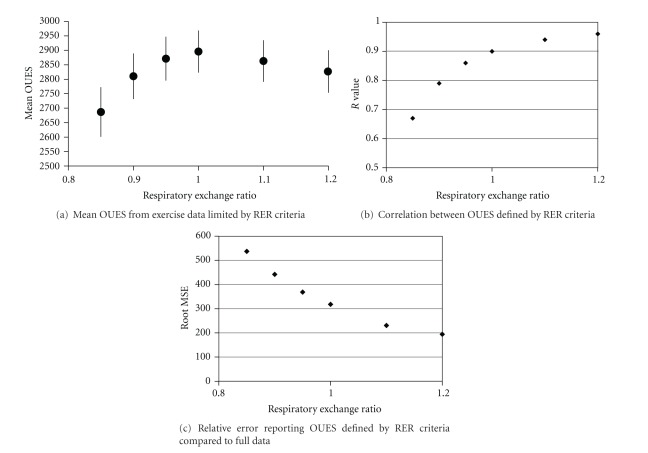
OUES values reported from exercise intensity defined by RER with associated correlation and relative error compared to full test data. Mean values are reported with standard error bars. (a) presents mean values of OUES generated from data associated with increasing RER. (b) presents the *R* value from Pearson Correlation comparing submaximal RER-defined exercise data with complete data. (c) shows the root mean squared error (RMSE) from the regression of the submaximal RER data versus complete exercise data. RMSE is presented as a value of the relative error reporting submaximal values as opposed to values from full test data.

OUES values from complete test data were strongly correlated with Peak VO_2_ (*r* = 0.86, *P* < 0.0001). OUES values were not independent of exercise intensity. The exercise test had to progress beyond 50% of the max duration and the ventilatory anaerobic threshold before OUES values approached that generated from full test data. Compared to full test data, the correlation increased and the error reduced for OUES values in association with increasing RER [Figures 2(a)–2(c)]. Using root mean squared error as a relative indicator of accuracy when reporting submaximal OUES, there is over 40% improvement reporting values from intensity corresponding with RER 1.0, compared with RER 0.85 (Figure 1). In the current study, OUES values peak in association with an RER of 1.0 (Table 2, Figure 1).

VAT Ventilatory Anaerobic Threshold, RER respiratory exchange ratio. Percentage of test defined by time to complete full test.

## 4. Discussion

Using peak and maximal exercise tests, OUES has been shown to be a valid, reproducible parameter of cardiopulmonary function and prognosis [10]. It is believed that OUES parameterises the relationship between peripheral oxygen demand and the associated increases in cardiac output and alveolar ventilation. In a noninvasive setting, it provides a composite value for the efficiency of the cardiopulmonary system to oxygenate and perfuse peripheral tissues and subsequent oxygen utilisation [10].

Recent OUES literature explores the test-retest validity and functional outcomes in new clinical cohorts reporting favourable results [20, 21]. This is supporting a potential transition in preoperative and respiratory clinical practice to use OUES in preference to the ventilatory anaerobic threshold. However, the use of OUES largely remains confined to the research field. This may change if the value and reliability of OUES as a submaximal measure is reported in the context of secondary reporting criterion. In noninvasive exercise testing, clinicians and physiologists commonly use predetermined criteria including heart rate percentages, RER, and visual analogy scores to validate the test. However, there are no established criteria for reporting OUES from submaximal tests.

**Figure 3:**
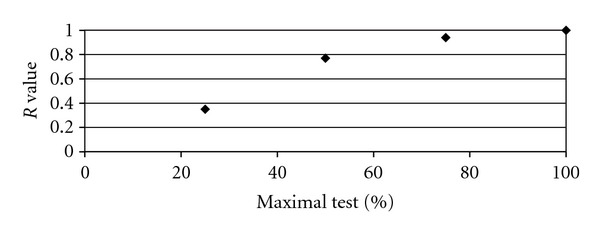
Correlation between submaximal and full test OUES values reported from percentiles of test data.

The stability of OUES in submaximal ranges has been noted since it was first introduced in the literature [9]. This understanding has expanded to recognise that OUES from 50% of a completed test is comparable to the full test OUES in both healthy and noncyanotic disease groups [22]. Davies et al. were the first to report that OUES from the first 50% of a modified Bruce protocol exercise test could be used as a prognostic indicator in heart failure [13]. This was subsequently confirmed by Arena et al. [14]. In these studies the difference between OUES from the first 50% of a maximal test and full test OUES was 1% and 2.6%, respectively. In the current study, the difference was 7.5% between full test OUES and OUES from 50% of a complete test. Not surprisingly, the difference between OUES and submaximal measures increased with increasing truncation of data. OUES values reported using data from the first quarter of the test could be as much as 35% lower than the highest values and correlation with full test values was low (*r* = 0.35) (Figure 3).

 The current study has presented the characteristics of OUES in relation to RER values. Using RER criteria, the OUES has been identified to peak at submaximal intensities. The highest mean OUES value occurs in association with an RER of 1.0.

This is consistent with the reported patterns of oxygen uptake efficiency using other parameters. Sun et al. identified that the highest values for the ratio of minute ventilation to oxygen uptake (VE/VO_2_) occurred in submaximal ranges [11].

**Figure 4:**
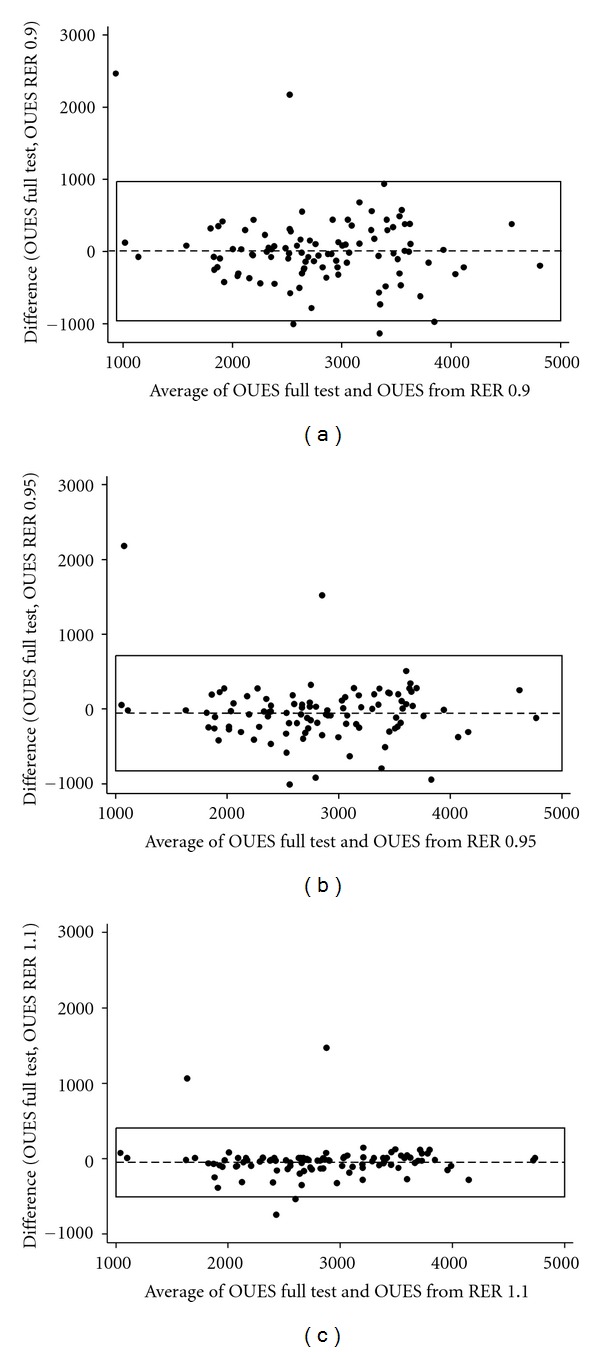
Bland Altman plots comparing full test data and data truncated using RER 0.9, 0.95, and 1.1 as cutoff criteria.

Using RER criteria, there is less than a 3% difference between OUES values reported from full test data and data from start of test to RER 1.0 (Table 2). Bland-Altman plots help to visualise the relationship between complete data OUES and results from RER 0.9, 0.95, and 1.1 (Figure 4). Mean difference between OUES from complete data and data up till RER 0.9 was less than 0.2% (3.86) with a correlation *r* value of 0.79. However, it should be noted that the limits of agreements contracted by over 50% when reporting OUES from RER 1.1 (limits −505 to 407) compared to using RER 0.9 (limits −961 to 969). Similar findings were reported by Van Laethem et al. when considering submaximal percentile data. Van Laethem also identified that test-retest reliability increased when OUES was calculated from peak exercise compared to submaximal [15]. Therefore, there are thresholds of reliability and reproducibility for reporting submaximal OUES values. In the current study, reporting OUES as RER increases above 0.9 provides values more reflective of the full test value. 

### 4.1. The Validity of RER as Secondary Criterion for Reporting OUES

At rest and low intensity exercise, there are multiple extrinsic determinants of the RER ranging from dietary intake to previous exercise load. However, the determinants of RER become intrinsic to exercise-induced metabolism as intensity increases between 25% and 70% of peak work rate [23].

In the current study, phase 1 exercise (level walking) generated a mean RER of 0.85 at a relative intensity of 40% of peak exercise (mean VO_2_ 16.0 mL/kg/min sd 2.4), while phase 3 (level jogging/running) generated a mean RER of 0.94 reaching a relative intensity of 71% peak VO_2_ (mean VO_2_ 28.4 mL/kg/min sd 3.5). These results are comparable to those of Goedecke who reported RER values of 0.86 (sd 0.037) and 0.976 (sd 0.043) during steady state 25% and 70% peak work rate (41% and 80% VO_2_ Peak) [23]. This would suggest that provided exercise intensity progresses beyond 40% of the predicted peak value of VO_2_ that RER can be used as valid criterion for identifying moderate intensity exercise.

RER is commonly used as a secondary criterion for satisfying the attainment of Peak VO_2_. This has recently been challenged with reports that there could be as much as a 27% difference in the Peak VO_2_ values recorded between an RER of 1.1 and maximal data [24]. The current study would suggest that the same concerns do not exist for reporting OUES. There is less than a 3% difference in comparison of the mean values across the RER range from 0.9 to 1.2 (Figure 2(a)).

In the current study, four individuals (4%) had peak RER less than 1.0 and there were a total of 16 individuals with peak RER values less than 1.1. It could be argued that these 16 individuals did not achieve true peak exercise. This prompted a review of the exercise characteristics of this group. Mean test duration completed by the group was 1087 seconds (sd 149). Although this test duration is lower than the full study group, all these low peak RER individuals continued to exercise beyond the start of phase 3 and 5 individuals entered the final stage of the protocol. The mean VAT of the group was 1516 mL/min (sd 509); this was not significantly different from the group with peak RER > 1.1. From the graphical data, 7 of the 16 individuals in this lower peak RER group attained a plateau in the VO_2_ curve including the individual with the lowest peak RER. The exercise characteristics of this subgroup do not raise immediate concern about the validity of these test results. They represent the normal distribution of peak RER identified by Goedecke et al. [23] and highlight the difficulties of defining maximal tests under noninvasive conditions.

**Figure 5:**
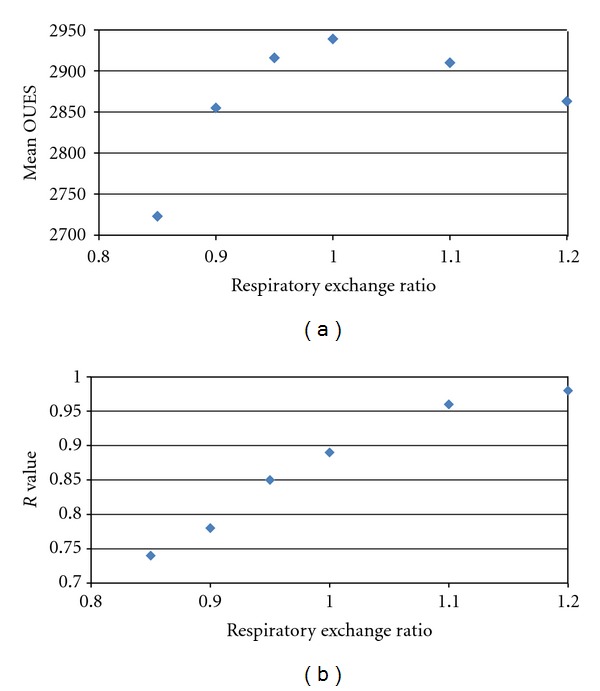
Subgroup analysis of 84 individuals with Peak RER > 1.1. (a) Mean OUES from exercise data limited by RER criteria. Group data for 84 individuals with peak RER > 1.1. (b) Correlation between OUES defined by RER criteria. Group data for 84 individuals with peak RER > 1.1.

As a sub-analysis the study group was divided into three groups dependent on the peak RER: group 1 peak RER less than 1.1 (*n* = 16), group 2 peak RER 1.1 to 1.2 (*n* = 42), and group 3 peak RER > 1.2 (*n* = 42). Statistical analysis of the group OUES values identified no significant differences between the group means. Group 1 mean OUES 2711 (sd 970), Group 2 mean OUES 2742 (sd 688), and Group 3 mean OUES 2926 (sd 636). Combining groups 2 and 3 to represent a maximal group (peak RER > 1.1), a further sensitivity analysis was performed identifying the same patterns as Figures 2(a) and 2(b) with regards to change in mean OUES with RER. OUES peaks in association with RER 1.0 (mean 2939 sd 682) with an associated *r* value of 0.89 compared to full test results, reflecting the pattern from the whole group (Figures 5(a) and 5(b)).

These subanalyses show that maximal OUES values do not vary significantly with the normal distribution of peak RER in a fit healthy cohort. There are not the same concerns about reporting OUES using RER criteria as there is a reporting peak VO_2_. The normal distribution of RER may raise concern that low peak RER individuals would be excluded if applying threshold exclusion criteria. However, in the present study only one individual had a peak RER less than 0.97. Further research is required to explore the characteristics and determinants of peak OUES at submaximal intensities; the highest value in this study occurred at RER 1.0 not with maximal data. The clinical relevance of using the highest value of OUES has not been explored. One of the rationales for using OUES is that it provides a composite value of cardiopulmonary fitness from across the spectrum of exercise intensity. Therefore, the assumption is that OUES reported from full test data would remain superior as a prognostic marker. These considerations require further formal investigation using invasive physiological measures.

### 4.2. Limitations

This study explored the validity of reporting submaximal values for OUES in healthy adults. It provides an indication of the variability in submaximal reporting, but it is limited by not being inclusive of a disease population. The relatively high peak VO_2_ suggests that the sample is biased towards inclusion of fitter individuals. It could be argued that using RER as reporting criterion with submaximal data would need a prospective validation within a disease population.

The RER levels defining the submaximal criteria were arbitrarily selected. Mean RER in the first 25% of the test was not below 0.85. To accurately define the cascade of physiological events in association with OUES, invasive monitoring and sampling would be required.

When combined with a rest test, the treadmill test protocol was designed to be representative of the intensity spectrum of physical activity encountered in daily living. The rise in intensity (ramp slope) is slower in comparison to other established protocols. This may affect the validity of the results; however, gas exchange patterns on the Wasserman plots were consistent with protocols of shorter test duration [8].

## 5. Conclusion

Cardiopulmonary exercise testing is a valuable research and clinical tool. Sub-maximal exercise testing produces a discrete set of limitations on the parameters that can be reliably reported. The ventilatory anaerobic threshold is often the preferred submaximal parameter. However, using VAT introduces potential observational error and can be time consuming to validate. The oxygen uptake efficiency slopes are validated as a prognostic indicator of cardiopulmonary disease that has the advantage of being objectively derived.

Previous studies reported that OUES can be regarded as independent of exercise intensity. This study identifies that there are certain caveats to that statement. OUES values change significantly during the transition from low to moderate intensity. There are definable reliability thresholds above 40% of peak exercise intensity, above 50% of time defined test, and in proximity of the VAT. During a ramp exercise protocol using RER as a reporting criterion, the current results predict higher confidence and reliability in an OUES value reported above an RER threshold of 1.0

## Figures and Tables

**Table 1:** Gas exchange parameters using the complete test to exhaustion data (peak exercise test data).

Variable	Mean	Standard deviation
Peak RER	1.19	0.11
Peak VO_2_ mL/min	2796	783
Peak VO_2_ mL/kg/min	39.8	8.8
Peak VO_2_ as percentage of predicted VO_2_max	131.5	20.4
VAT mL/kg/min	23.6	4.5
OUES mL/min per 10-fold ventilation increase	2814	718
OUES mL/min/kg per 10-fold ventilation increase	40.1	8.2
% Predicted OUES	130.7	34.2
VE/VCO_2_ slope	30.2	4.15
Predicted VE/VCO_2_ slope	30.6	1.56

Predicted values for VO_2_max, OUES, and VE/VCO_2_ slope generated from prediction equations. Abbreviations: RER, respiratory exchange ratio; VO_2_, oxygen consumption; VE, minute ventilation; VCO_2_, carbon dioxide production; VAT, ventilatory anaerobic threshold; OUES oxygen uptake efficiency slope.

**Table 2:** OUES values generated from restricting data points by percentile, respiratory exchange ratio, and ventilatory anaerobic threshold.

Submax criterion	Mean OUES	Standard deviation
Percentile of test data		
0–25%	1875	545
0–50%	2604	750
0–75%	2832	667
Respiratory exchange ratio		
<0.85	2687	860
<0.90	2810	789
<0.95	2871	759
<1.0	2895	730
<1.10	2863	719
<1.20	2824	734
Up to ventilatory anaerobic threshold	2672	687
Complete test data	2814	718
